# Hemoptysis due to ruptured lung abscess successfully treated by exploratory thoracoscopy: report of a case

**DOI:** 10.1186/s44215-023-00105-7

**Published:** 2023-08-25

**Authors:** Yutaka Miyano, Kunihiro Oyama, Yuka Saito, Masato Kanzaki

**Affiliations:** 1 Department of Thoracic Surgery, Saiseikai Kazo Hospital, Kamitakayanagi 1680, Kazo-Shi, Saitama, Ken 347-0101 Japan; 2 Department of Respiratory Medicine, Saiseikai Kazo Hospital, Kamitakayanagi 1680, Kazo-Shi, Saitama, Ken 347-0101 Japan; 3 Department of Thoracic Surgery, Tokyo Women’s Medical University, Kawada-Cho 8-1, Shinjuku-Ku, Tokyo, 162-8666 Japan

**Keywords:** Ruptured lung abscess, Hemoptysis, Exploratory thoracoscopy

## Abstract

**Background:**

Ruptured lung abscess is rare and a serious medical concern. We reported a rare case of exploratory thoracoscopy for hemoptysis and empyema due to ruptured lung abscess.

**Case:**

A 49-year-old man visited our hospital due to hemoptysis. Chest CT showed a left empyema due to ruptured lung abscess in the left lower lobe. Exploratory thoracoscopy revealed thoracic empyema with bronchopleural fistula, and thoracotomy was performed and curettage for empyema cavity, and closure of bronchopleural fistula was done. It was closed by sutures with polyglycolic-acid sheet as a pledget and covered with thickened pleura. A pneumonia and a small cavity, which seemed to be aspiration, were found on the healthy side, and the patient is being closely followed up.

**Conclusion:**

We encountered a surgical case of ruptured lung abscess that is a cause of hemoptysis, and postoperative course was uneventful. Exploratory thoracoscopy was effective in confirming the fistula.

## Background

There are few reports of hemoptysis caused by ruptured lung abscess, which is a serious medical condition. We report here a case of ruptured lung abscess with hemoptysis and empyema, which healed with surgical treatment after exploratory thoracoscopy, along with a brief discussion.

## Case report

A 49-year-old man consulted our hospital because of hemoptysis. There was no history of hematologic, malignant, or inflammatory disease, only with disc herniation.

In December 2021, the patient visited a local physician with a chief complaint of bloody sputum and was planned to be referred to the department of respiratory medicine of our hospital for suspected carcinomatous pleurisy and acute empyema. Two days later, however, the patient coughed up a cupful of blood and returned to the physician, and the patient was referred to our department.

### Clinical findings on admission

The body temperature was 37.7 °C, blood pressure 130/86 mmHg, and oxygen saturation as measured by pulse oximetry (SpO_2_) 96% (while breathing ambient air). There were no palpable superficial lymph nodes. The heart sounds were regular, with no murmur. The breath sounds were diminished on the left side.

The patient had a significantly increased inflammatory reaction (WBC 21,070/mm^3^ and *CRP* 20.45 mg/dL), a reduced albumin level (Alb 2.0 g/dL), an anemia (Hb 10.3 g/dL), and an impaired glucose tolerance (Glu 152 mg/dL and HbA1c 6.3%).

### Imaging findings

**Fig. 1:**
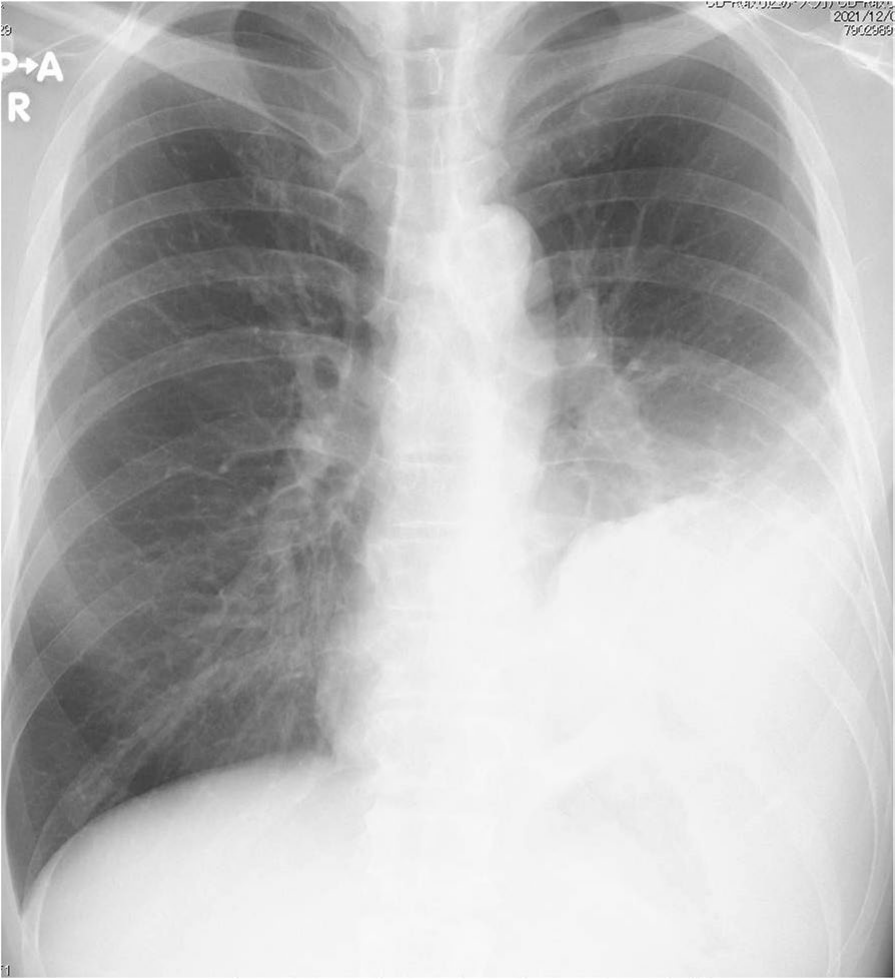
Chest x-ray showing dense opacity pleural effusion in the lower left lung fields

**Fig. 2:**
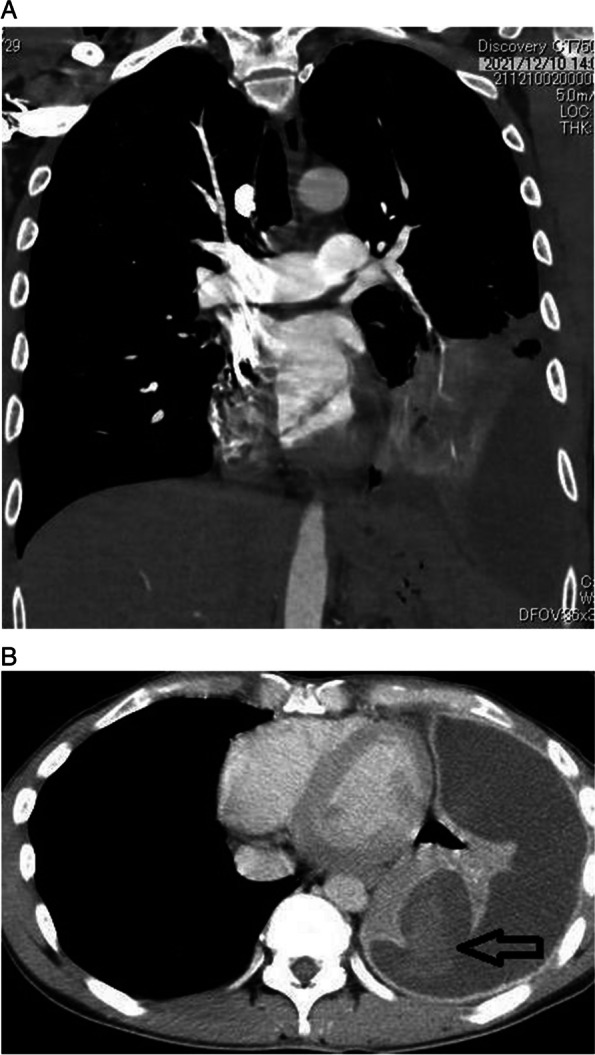
**a** Chest CT showing a left empyema due to ruptured lung abscess in the left lower lobe. **b** A pale high-concentration hematoma is suspected in the perforation (arrow), but no obvious extravasation is observed

**Fig. 3:**
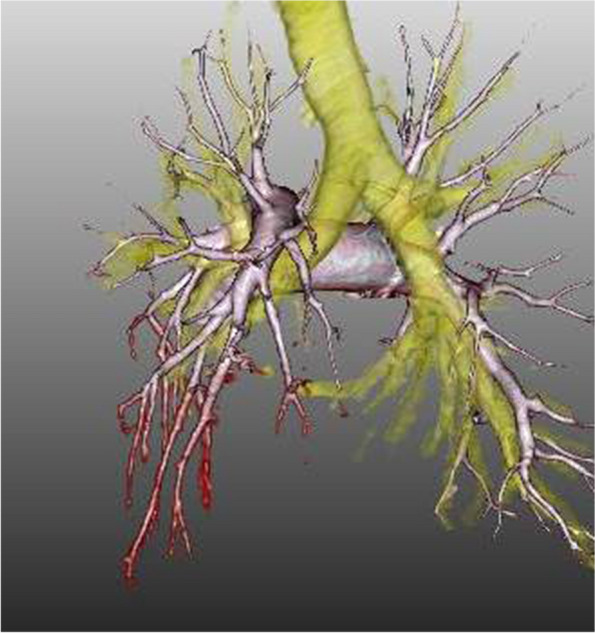
3D CT could identify no findings that cause bleeding, such as lung cancer, pulmonary aneurysm, and arteriovenous malformation

Chest radiography (Fig. 1) revealed an area of decreased lucency in the left lower lung field, for which fluid accumulation was suspected. Chest computed tomography (Fig. 2a) revealed an extensive abscess in the left lower lobe perforating into the thoracic cavity to form acute empyema. Although a hematoma as a slightly high-density area was suspected at the perforated area (Fig. 2b), there was no evidence of extravasation. Three-dimensional computed tomography revealed no findings suggestive of the cause of bleeding, such as lung cancer, pulmonary artery aneurysm, or arteriovenous malformation (Fig. 3).

### Findings of exploratory thoracoscopy and surgery

**Fig. 4:**
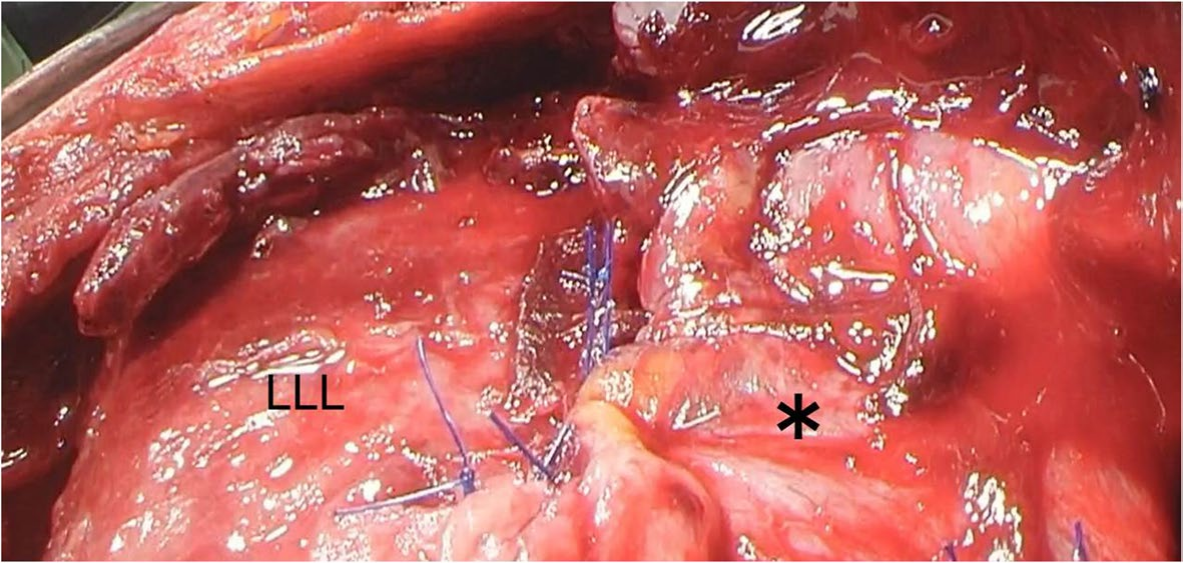
The bronchial fistula was closed by suturing Neoveil® as a cotton ball and then covering the sutured area with a thickened pleura (*). LLL, left lower lobe

A diagnosis of hemoptysis and empyema due to ruptured lung abscess was made, and exploratory thoracoscopy was performed. Thoracoscopic observation using a port placed on the seventh rib midaxillary line revealed a significant leakage and identified the perforated area discharging pus in the left lower lobe. Thoracotomy along the eighth rib was performed with an additional skin incision, followed by pleural lavage and closure of bronchial fistula. One of the aims of surgery was to prevent massive hemoptysis, so lobectomy was also considered. However, there was an abscess in the thoracic cavity and no hemothorax, and the inflammation was at its extreme stage, making lobectomy difficult. No intra-abscess hematoma was observed, suggesting that hemoptysis occurred only at the time of perforation. Therefore, we decided to perform intrathoracic lavage and bronchial fistula closure. The ruptured lung abscess was completely drained, allowing lavage and bronchial fistula closure. The surgical finding was acute empyema, and sufficient lung expansion was obtained. We judged that it was possible to control the infection and considered that the primary purpose was to close the fistula and avoid chronic empyema with a fistula. The fistula was closed by suture using a polyglycolic-acid (PGA) sheet (Neoveil®, Gunze, Kyoto, Japan) as pledgets and covered by thickened parietal pleura with pedicles (Fig. 4).

### Postoperative course

**Fig. 5:**
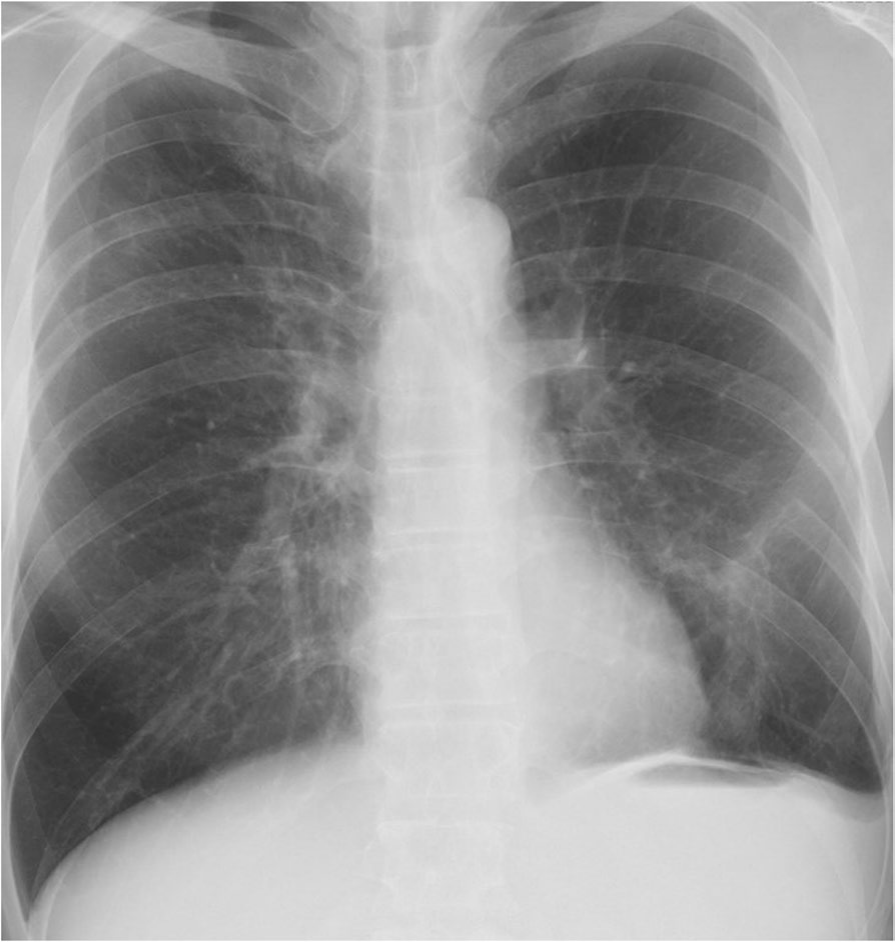
Left empyema and right pneumonia and cavity also healed

The surgery lasted 3 h, with a bleeding of 99 g and aspirated pus of 750 g. *Acinetobacter baumannii* was isolated from a pus culture. The patient had no postoperative complications, and thus, the drain at the apex was removed on day 12 of illness. On postoperative day 4, radiographic features of contralateral pneumonia and small cavity presumably due to aspiration were observed. On day 34 of illness, the patient was discharged from the hospital, with the basal drain left for a persistent minor leakage. Subsequently, the leakage resolved, and the features of contralateral pneumonia and small cavity also improved (Fig. 5), and the basal drain was removed on day 64 of illness. Antibiotics were administered daily for 39 days of hospitalization and, after discharge, were administered in outpatient clinics twice a week until postoperative day 68. No relapse of empyema or air leakage has been observed as of 6 months after surgery.

## Discussion

There are very limited reports of ruptured lung abscess which caused hemoptysis. Approximately, 10% of the hemoptysis cases are attributable to empyema, and the majority of them are due to the rupture of vessels or bronchial artery from the chest wall in chronic empyema [1]. In addition, only 1 to 3% of empyema cases are attributable to ruptured lung abscess [2]. Whereas adult patients with empyema mostly have some serious underlying diseases, [3] our patient had no underlying disease or history of smoking. As the causative bacteria, *A. baumannii* was isolated from the intraoperative pus culture.

Infections with *A. baumannii*, accounting for approximately 80% of the cases of *Acinetobacter* infections, tend to occur in hospitalized patients with severe disease. Whereas infections with *A. baumannii* most frequently occur in the respiratory system, these can cause purulent infections in any organ systems [4]. Respiratory infections with *A. baumannii* have been reported to occur as nosocomial pneumonia, fulminant community-acquired pneumonia, chronic community-acquired pneumonia, and some other forms. Fulminant community-acquired pneumonia due to *A. baumannii* is characterized by severe conditions frequently accompanied by septic shock and death [4, 5]. Our patient also probably suffered from pneumonia with secondary pulmonary abscess.

The general treatment of empyema is aimed at clearing and reducing the affected thoracic area with antibiotic therapy and chest drainage. However, in empyema with fistula due to ruptured lung abscess, puncture and drainage may cause contralateral aspiration. In our case, there was no niveau formation on the image, no air space in the thoracic cavity, and no aspiration image in the lung on preoperative CT, and the fistula was occluded by the contents of the abscess. Therefore, we proceeded with preparations for examination thoracoscopy without drainage. According to Hagan et al., surgery is indicated for lung abscess complicated by bronchial fistula and/or empyema if the patient’s overall condition allows [2]. In cases of ruptured lung abscess, almost always accompanied by acute empyema with fistula, surgery should always be considered if the patient’s overall condition allows. An operative procedure should be determined based on the size and location of ruptured abscess cavity and the condition of bronchial fistula. In reported Japanese cases, thoracotomy and video-assisted thoracoscopic surgery were performed. In surgery for ruptured lung abscess, curettage of empyema cavity and drainage for acute empyema should be performed first, followed by the treatment of ruptured abscess cavity and resultant bronchial fistula. For abscess cavity and bronchial fistula, exploratory thoracoscopy should be considered if the abscess cavity is very large or bronchial fistula may be present, which is difficult to close by suture [6]. In our patient, closure of bronchial fistula was performed because the abscess cavity and the identified bronchial fistula were large, and lobectomy was not feasible because of the most advanced inflammation. Because the tissue was fragile, the fistula was closed by suture using Neoveil® as pledgets and covered by thickened parietal pleura and a tissue sealing sheet (TachoSil®, CSL Behring, PA, USA).

Ruptured lung abscess, which is a rare medical condition, may require exploratory thoracoscopy for selecting an operative procedure for empyema with fistula.

## Concluding remarks

We encountered a patient with lung abscess perforating into the thoracic cavity, whose postoperative course was uneventful. Exploratory thoracoscopy was effective in identifying the site of perforation and selecting the operative procedure.
