# Correction: Hemoptysis due to ruptured lung abscess successfully treated by exploratory thoracoscopy: report of a case

**DOI:** 10.1186/s44215-023-00111-9

**Published:** 2023-11-20

**Authors:** Yutaka Miyano, Kunihiro Oyama, Yuka Saito, Masato Kanzaki

**Affiliations:** 1 Department of Thoracic Surgery, Saiseikai Kazo Hospital, Kamitakayanagi 1680, Kazo-Shi, Saitama, Ken 347-0101 Japan; 2 Department of Respiratory Medicine, Saiseikai Kazo Hospital, Kamitakayanagi 1680, Kazo-Shi, Saitama, Ken 347-0101 Japan; 3 Department of Thoracic Surgery, Tokyo Women’s Medical University, Kawada-Cho 8-1, Shinjuku-Ku, Tokyo 162-8666 Japan

**Correction: Gen Thorac Cardiovasc Surg Cases 2, 86 (2023)**

**https://doi.org/10.1186/s44215-023-00105-7**

Following publication of the original article [1], we have been notified that Competing interests’ section was published incorrectly as per below:

The authors declare that they have no competing interests.

It should be:

MK is the Editor of General Thoracic and Cardiovascular Surgery Cases. The other authors declare that they have no competing interests.
